# Prevalence of Cerebrovascular Accidents Among the US Population With Substance Use Disorders: A Nationwide Study

**DOI:** 10.7759/cureus.31826

**Published:** 2022-11-23

**Authors:** Nikhila Chelikam, Zeeshan Mohammad, Krishna Tavrawala, Anjali N Krishnakumar, Anitta Varghese, Tanvi Yogesh Shrivastav, Baris Tarimci, Sushil Kumar, Stephan Z Francis, Vikramaditya Samala Venkata, Urvish K Patel, Lokesh Manjani

**Affiliations:** 1 Internal Medicine, Icahn School of Medicine at Mount Sinai, New York, USA; 2 Internal Medicine, BLK-Max Super Speciality Hospital, New Delhi, IND; 3 Internal Medicine, Smt. B. K. Shah Medical Institute & Research Centre, Vadodara, IND; 4 Internal Medicine, Ahalia Diabetes Hospital, Palakkad, IND; 5 Emergency Medicine, Iqraa International Hospital and Research Centre, Kozhikode, IND; 6 General Medicine, Davao Medical School Foundation, Davao City, PHL; 7 Internal Medicine, Ege University Faculty of Medicine, Izmir, TUR; 8 Internal Medicine, Mallige Hospital, Banglore, IND; 9 Internal Medicine, Saba University School of Medicine, The Bottom, BES; 10 Internal Medicine, Cheshire Medical Center, Dartmouth-Hitchcock, Keene, USA; 11 Public Health and Neurology, Icahn School of Medicine at Mount Sinai, New York, USA; 12 Internal Medicine, Medsrar Washington Hospital Center, Washington, DC, USA

**Keywords:** prevalence, epidemiology and public health, cross-sectional study, nhanes, substance use, stroke

## Abstract

Introduction

Globally, stroke is one of the top ten causes of death. The incidence of stroke in patients aged 44 years and younger was noted to have risen over the past three decades. This rise in stroke diagnosis among young adults could be attributed to multiple reasons, including the rising prevalence of comorbidities like diabetes, hypertension, substance use disorders (SUDs), etc.

Aim & objectives

This study's primary aim was to evaluate the prevalence of stroke in the US population and the prevalence of SUDs amongst patients with a prior history of stroke. The secondary aim was to evaluate the association between Stroke and SUDs.

Methods

Our population was obtained from the National Health and Nutrition Examination Survey (NHANES) between the years 2013 to 2018. We identified respondents diagnosed with stroke using the questionnaire and the history of various SUDs amongst this population. The data were analyzed using SAS software (Version 9.4). We performed univariate analysis using the chi-square and Mann-Whitney test, and a p-value of <0.05 was considered statistically significant.

Results

Two hundred sixty-four thousand seven hundred forty (264,740) respondents were included in this study, and 10435 (3.94%) respondents were noted to have a history of stroke. The population subset with a stroke diagnosis was older (68 years vs. 51 years). Higher prevalence was noted among the female sex (52.14% females vs. 47.86% males), Non-Hispanic white ethnicity, followed by Non-Hispanic black & then other Hispanics (47.56% vs.25.47% vs. 7.82%), and those belonging to a lower annual household income of $0-$25,000 and $25,000-$65,000 ( 46.61% vs. 35.93% ). (p<0.0001). After adjusting for socio-demographics and coexisting comorbidities, e-cigarette [OR: 2.03; 95% CI: 2.03-2.03], cocaine [OR: 1.54; 95%CI:1.54-1.54], heroin [OR: 1.83; 95%CI: 1.83-1.83], marijuana or hashish [OR: 1.01; 95% CI: 1.01-1.01], were observed to have an association with higher odds of stroke than the population without a history of using these illicit drugs.

Conclusion

Among respondents with a history of stroke, the use of cocaine was most prevalent, followed by marijuana/hashish, heroin, e-cigarettes, and injecting illegal drugs. The odds of having a stroke were two times higher in the population using an e-cigarette and higher among those using heroin, cocaine, and marijuana/ hashish. The Government should plan policy changes to treat SUDs in the USA, which could help reduce the stroke burden. Recall that bias and geographic variations in response rate by participants of the study were the limitations of our survey-based study.

## Introduction

Stroke is one of the leading causes of mortality and morbidity worldwide [1]. While it is predominantly seen in the elderly, the incidence of stroke in younger populations less than 44 years old is rising. Between 1995 to 2011, the hospitalization rate for stroke in this subset of the population almost doubled [2]. This change can be attributed to an increase in the prevalence of comorbidities such as diabetes and hypertension [3] and a rise in rates of substance abuse disorders (SUDs) among young adults [4]. Drug use is among the most common and significant predisposing conditions for stroke among people under 35 years of age [5]. Substance use was revealed to be the fifth most prevalent cause of ischemic stroke in patients aged 18 to 44, according to the Baltimore- Washington Cooperative Young Stroke Study [6]. 

Among the different illicit substances, stimulant drugs have been shown to relate to higher stroke and stroke-related mortality rates. Westover et al. reported the highest rates of stroke diagnosis in users of amphetamines, followed by cannabis and cocaine [4]. Amphetamine abuse was also associated with a higher risk of death after a hemorrhagic stroke [4]. In the United States, cocaine is one of the most commonly abused drugs, and about 4.7 million Americans aged 12 and above had used cocaine in 2013, and nearly 38 million had consumed the substance at some point [7]. Cocaine has been well-reported as a contributing factor to hemorrhagic and ischemic strokes [8-10]. Cannabis has more than 120 million users globally, making it the most widely used substance among others [11]. This rise can be attributed to the legalization of cannabis for both medical and recreational purposes [12]. However, only a little has been put forward about the harmful effects of cannabis. Multiple case reports and epidemiological studies reported the association between marijuana use and ischemic stroke, particularly in young adults [4,12-14].

Other than SUDs, alternative modifiable risk factors for stroke in this population include hypertension, hyperlipidemia, diabetes mellitus, coronary heart disease [15], smoking [15-17], heavy alcohol consumption, low physical activity [15,16], and obesity [16,18]. We aim to look at the prevalence of SUDs amongst the stroke population and the relationship between stroke and various SUDs.

## Materials and methods

Details of Data

Data were obtained from the National Health and Nutrition Examination Survey (NHANES), a cross-sectional population-based survey intended to evaluate the health of children and adults in the USA, directed by the Centers for Disease Control and Prevention (CDC). NHANES data are declared in 2-year cycles and use a multistage probability sampling pattern to create a nationally depictive sample. The sampling design and protocol of NHANES are analyzed by the US Department of Health and Human Services and approved by the National Center for Health Statistics Research Ethics Review Board annually. The NHANES surveys comprehend demographic, socioeconomic, dietary, laboratory tests, and health-related questions. The clinical examination consists of medical, dental, and physiological measurements and laboratory tests conducted by exceptionally trained medical field persons. The dataset information and user guide are available on the CDC website https://wwwn.cdc.gov/nchs/nhanes/Default.aspx.

Study Population and Definitions

A retrospective cross-sectional study based on the NHANES database between the years 2013 to 2018 was conducted. The individual datasets were downloaded and then combined using Version 9.4 SAS software. For weighting multiple years of NHANES data, appropriate weighting procedures were employed. We included participants of age 18 years and above, diagnosed with a stroke, and who had complete data from the NHANES Drug Use questionnaires. Sociodemographic variables such as age, gender, race, and annual household income, and comorbidities such as coronary heart disease, hypertension, congestive heart failure, diabetes mellitus, dyslipidemia, liver disorder, alcohol use, drug use, depression, cancer or malignancy, and lab values such as LDL cholesterol and HbA1c levels were included for this study. Participants were excluded if there was any missing information about their age, race, stroke, and drug use.

Stroke: Patients diagnosed with stroke are assessed by questions: MCQ160f: "Has a doctor or other health professional ever told {you/SP} that {you/s/he} . . .had a stroke?"

Heavy Alcohol Use: Heavy Alcohol use was categorized using the following question: ALQ151: Ever have 4/5 or more drinks daily? - Was there ever a time or times in {your/SP's} life when {you/he/she} drank {DISPLAY NUMBER} or more drinks of any alcoholic beverage almost every day? According to the Centers for Disease Control and Prevention, Excessive alcohol use includes binge drinking, defined as five or more drinks on occasion (within two or three hours) for men and four or more drinks on occasion (within two or three hours) for women. Other forms of excessive alcohol use include heavy drinking (15 or more drinks a week for men, eight or more drinks a week for women),

Substance Use Disorder (SUDs): SUDs were assessed using the following questions: DUQ200: "The following questions ask about the use of drugs not prescribed by a doctor. Please remember that your answers to these questions are strictly confidential. The first questions are about marijuana and hashish. Marijuana, also called pot or grass, is usually smoked in cigarettes, joints, or pipes and sometimes cooked in food. Hashish is a form of marijuana that is also called 'hash.' It is usually smoked in a pipe. Another form of hashish is hash oil. Have you ever, even once, used marijuana or hashish?"; DUQ 250: "The following questions are about cocaine, including all the different forms of cocaine such as powder, 'crack,' 'free base,' and coca paste. Have you ever, even once, used cocaine in any form?"; DUQ 290. "The following questions are about heroin. Have you ever, even once, used heroin?"; DUQ330 "The following questions are about methamphetamine, also known as crank, crystal, ice, or speed. Have you ever, even once, used methamphetamine?"; DUQ370: "The following questions are about the different ways that certain drugs can be used. Have you ever, even once, used a needle to inject a drug not prescribed by a doctor?" 

Demographic characteristics in the analysis included age, gender, race, and annual household income at the time of the survey's conduction. These variables were obtained by asking the participants, Are you Male or Female? And how old are you? Race/ethnicity was classified as Mexican American, Hispanic, other Hispanic, non-Hispanic white, non-Hispanic black, non-Hispanic Asian, and Other Race. 

The comorbidities used in this study were coronary heart disease, hypertension, congestive heart failure, diabetes mellitus, dyslipidemia, liver disorder, depression, cancer, or malignancy and were assessed by those who answered yes to the questions: Have you EVER been told by a doctor or other health professional that you had… (1) coronary (kor-o-nare-ee) heart disease? (2) hypertension, also called high blood pressure +2 times, (3) congestive heart failure, (4) diabetes or sugar diabetes? (5) high cholesterol, (6) any liver condition, (7) depression, (8) cancer, or a malignancy of any kind? Respectively. People who refused were not asked or did not know coded as missing.

Aims

This study's primary aim was to evaluate the prevalence of stroke in the US population and the prevalence of substance use disorder (SUD) amongst patients with a history of stroke. The secondary aim was to evaluate the association between stroke and substance use.

Statistical Analysis

Using Version 9.4 of SAS software, the data was analyzed. Univariate analysis was performed to find the association between SUDs, stroke, and other sociodemographic variables using chi-square for categorical variables and the Wilcoxon Rank Sum test for continuous variables. To predict the association of different SUDs with stroke, multivariable survey logistic regression models were generated after adjusting for confounding variables to estimate the odds ratio (OR) and 95% Confidence Intervals. A p-value of <0.05 was considered statistically significant. 

## Results

Demographic and Co-morbidity characteristics

An overall population of 415,273 from 2013 to 2018 was narrowed down to 264,740 after excluding the pediatric population (age < 18 years old) and adults with missing data on age or gender. Out of 264,740, 10,435 (3.94%) had a history of stroke within their lifetime. Respondents who had experienced a stroke were older (68 vs. 51), and a pattern of higher prevalence was noted among females (52.14% females vs. 47.86% males; p<0.0001), Non-Hispanic white ethnicity, followed by Non-Hispanic black & then other Hispanics (47.56% vs.25.47% vs. 7.82%); p<0.0001), population belonging to a lower annual household income $0-$25,000 and $25,000-$65,000 (46.61% vs. 35.93%); p<0.0001). Among the demographic categories with higher prevalence, females who had experienced stroke were less prevalent than females without a history of stroke (52.14% vs. 52.67%). The non-Hispanic white population with a history of stroke was higher as compared to the former with no history of stroke (47.56% vs. 39.09%), and a majority of respondents with a history of stroke belonged to lower annual household income as compared to respondents with no history of stroke (46.61% vs. 26.26%). Concurrent prevalence of coronary heart disease (21.48% vs. 3.97%), congestive heart failure (18.37% vs. 2.94%), hypertension (87.97% vs. 79.91%), hypercholesterolemia (61.26% vs. 36.35%), diabetes (36.51% vs. 13.94%) and depression (9.12% vs. 2.77%) was higher amongst respondents with stroke in comparison to without stroke. (p<0.0001) (Table 1).

**Table 1:** Characteristics of stroke population from NHANES between 2013 to 2018 LDL: low-density lipoprotein;*Calculated by NIH equation 2 (mg/dl)

Variables	Stroke N=10435 (3.9%)	No Stroke N= 254305 (96.06%)	Total N= 264740 (100%)	p-value
Demographic and Socioeconomic Characteristics (%)				
Age in years at screening (Median + IQR)	68 (59-78)	51 (36-64)		< .0001
Gender (%)				0.2908
Female	5441 (52.14)	133939 (52.67)	139380 (52.65)	
Male	4994 (47.86)	120366 (47.33)	125360 (47.35)	
Race (%)				< .0001
Mexican American	945 (9.06)	36592 (14.39)	37537 (14.18)	
Other Hispanic	816 (7.82)	27098 (10.22)	27914 (10.54)	
Non-Hispanic White	4963 (47.56)	99398 (39.09)	104361 (39.42)	
Non-Hispanic Black	2658 (25.47)	49747 (19.56)	52405 (19.79)	
Non-Hispanic Asian	459 (4.40)	32249 (12.68)	32708 (12.35)	
Other Race - Including Multi-Racial	594 (5.69)	9221 (3.63)	9815 (3.71)	
Annual Household Income (AHI) (%)				< .0001
$0 - $25,000	4489 (46.61)	61895 (26.26)	66384 (27.26)	
$25,000 - $65,000	3461 (35.93)	84332 (36.06)	87793 (36.05)	
$65,000 - $100,000	905 (9.40)	36631 (15.66)	37536 (15.41)	
>$100,000	777 (8.07)	51030 (21.82)	51807 (21.27)	
Concurrent comorbidities (%)				
Coronary Heart Disease (%)	2241 (21.48)	10091 (3.97)	12332 (4.66)	< .0001
Congestive Heart Failure (%)	1917 (18.37)	7479 (2.94)	9396 (3.55)	< .0001
High Blood Pressure - 2+ Times (%)	6880 (87.97)	75405 (79.91)	82285 (80.53)	< .0001
Recent Systolic Blood Pressure in mmHg (Median)	136	137		< .0001
Recent Diastolic Blood Pressure in mmHg (Median)	80	80		< .0001
Taking Prescribed Medicine for HBP (%)	6770 (90.21)	71870 (86.47)	7864 0 (86.78)	< .0001
High Cholesterol Level (%)	6392 (61.26)	92436 (36.35)	98828 (37.33)	< .0001
Taking Prescribed Medicine High Cholesterol (%)	5350 (81.83)	52676 (74.39)	58026 (75.02)	< .0001
LDL-Cholesterol	96 (73-134)	110 (88-135)		
Diabetes (%)	3810 (36.51)	35461 (13.94)	39271 (14.83)	< .0001
Last Hb A1C Level (Median)*	8	7.8		
Feel Depressed nearly Every Day	846 (9.12)	6603 (2.77)	7449 (3.00)	< .0001
Liver Disorders (%)	876 (8.39)	12147 (4.78)	13023 (4.92)	< .0001

Prevalence and characteristics of SUDs

Use of marijuana/hashish [Stroke vs. No stroke; 63.72% vs. 52.97%; p<0.0001], traditional smoking [57.20% vs. 41.39%; p<0.0001], cocaine/heroin/methamphetamine [24.29% vs. 16.86%; p<0.0001], injectable drugs [4.21% vs. 2.49%; p<0.0001] were found to be prevalent amongst the respondents who had a positive history of stroke when compared to respondents with no prior history of a stroke. However, a reverse characteristic was observed with cocaine use [89.91% vs. 95.56%; p<0.0001], alcohol use disorder- heavy drinking [36.44% vs. 47.21%; p<0.0001], and e-cigarettes use [12.71% vs. 17.09%; p<0.0001] (Table 2).

**Table 2:** Prevalence of SUDs amongst the stroke population

Variables	Stroke N=10435 (3.9%)	No Stroke N= 254305 (96.06%)	Total N= 264740 (100%)	p-value
Traditional smoking (Current or >100 cigarettes)	5969 (57.20)	105249 (41.39)	111218 (42.01)	< .0001
E-cigarettes	921 (12.71)	28091 (17.09)	29012 (16.90)	< .0001
Alcohol use disorder – Heavy drinking (%)	1761 (36.44)	78113 (47.21)	79874 (46.91)	< .0001
Ever use of Marijuana or Hashish (%)	1591 (63.72)	80689 (52.97)	82280 (53.15)	< .0001
Ever use any form of cocaine	1105 (89.91)	31579 (95.56)	32684 (95.36)	< .0001
Ever use any form of Heroin	288 (23.43)	5028 (15.21)	5316 (15.51)	< .0001
Ever use any form of methamphetamine	563 (45.81)	13122 (39.71)	13685 (39.33)	< .0001
Inject Illegal Drug (%)	213 (4.21)	4882 (2.49)	5095 (2.53)	< .0001

Regression analysis showing an association between SUDs and Stroke

Following the adjustment for socio-demographics (such as age, gender, race, and annual household income) and concomitant comorbidities (such as hypertension, coronary heart disease, congestive heart failure, diabetes, hypercholesterolemia, and depression), e-cigarette [OR: 2.03; 95% CI: 2.03-2.03], heroin [OR: 1.83; 95%CI: 1.83-1.83], cocaine [OR: 1.54; 95%CI: 1.54-1.54], and marijuana or hashish [OR: 1.01; 95% CI: 1.01-1.01] were associated with higher odds of cerebrovascular disease or stroke among other SUDs. (p<0.0001) (Table 3).

**Table 3:** Multivariable survey logistic regression analysis showing the relationship between SUDs and Stroke The model was adjusted for socio-demographics (age, race, gender, and annual household income) and concurrent comorbidities (hypertension, coronary heart disease, congestive heart failure, diabetes, hypercholesterolemia, and depression).

Outcomes	Odds Ratio	95% CI	p-value
E-cigarette use	2.03	2.03-2.03	< .0001
Ever use of heroin	1.83	1.83-1.83	< .0001
Ever use of cocaine	1.54	1.54-1.54	< .0001
Ever use of Marijuana or Hashish	1.01	1.01-1.01	< .0001
Alcohol use disorder - Heavy drinking	0.91	0.91- 0.91	< .0001
Ever use of methamphetamine	0.80	0.80-0.81	< .0001
Traditional smoking (Current/ >100 cigarettes)	0.67	0.66-0.67	< .0001
Ever use of Inject Illegal	0.23	0.23-0.23	< .0001
c-value	0.79		

## Discussion

Stroke is a leading cause of mortality and morbidity, accounting for 1 of every 19 deaths and a significant cause of severe long-term disability in the United States [2]. The median stroke prevalence in United States Adults is 3%, according to Heart Disease and Stroke Statistics 2020 [2]. Our study showed a slightly higher prevalence at 3.94%. Even though there has been a rise in the incidence of stroke in the young population over the past three decades [19], stroke continues to be more prevalent in the elderly, with prevalence being highest in the age group >65 years [20]. Our study showed that the median age of the stroke population at 68, compared to 51 in the non-stroke population. According to heart disease and stroke statistics 2020, each year, 55000 more females have a stroke compared to males [2]. Females have been shown to have a higher lifetime risk of stroke than males [21]. In our study, the prevalence of stroke was more in females than males; however, this was not statistically significant.

Between the years 1995 to 2011, stroke-related hospitalizations in the age group 18 to 44 almost doubled [2]. Along with a rise in the prevalence of comorbidities like diabetes, and hypertension [3], SUDs are a leading cause of ischemic stroke in young adults [5,6]. The Baltimore Washington Cooperative Young Stroke Study carried out in several institutions across the US between 1988 and 1991 attributed illicit substance use as a significant cause of stroke among the youth [6]. In 2021, a population-based study of stroke among 450,000 citizens in Iran concluded that SUD raised the mortality risk among stroke patients [22].

In the United States, cocaine and marijuana are the most commonly used illicit drugs [7]. Our study showed similar results, with cocaine and marijuana being the most common illicit drugs used in stroke and non-stroke groups, along with methamphetamines. There are various reported mechanisms through which SUDs cause a stroke. Cannabinoid use could lead to ischemic stroke via transient vasospasm. Moreover, using opiates via the intravenous route can cause infective endocarditis, leading to ischemic stroke [23]. Multiple studies have shown an association between cocaine use and the risk of early-onset ischemic stroke [10,24,25]. Cocaine can lead to stroke even after using them once due to vasospasm and other unknown mechanisms [8,26]. The exact mechanism for cocaine-induced stroke has not been identified. However, several pathways have been postulated, including vasospasm, cerebral vasculitis, hypertensive surge, and enhanced platelet aggregation [8,25]. In our study, similar to prior studies, the odds of having a stroke were observed to be higher in users of cocaine. 

Current evidence is not clear regarding the association between stroke and marijuana use. While studies by Bayan Moustafa et al. and a few others showed a higher risk of stroke in marijuana users [4,27,28], others did not find any association between stroke and marijuana [29,30]. In our study, the use of marijuana was associated with higher odds of stroke. Similar to cocaine and marijuana use, our study also showed higher odds of developing a stroke with e-cigarette use and heroin use, consistent with existing evidence [31,32].

Existing data regarding the association between alcohol use and stroke is not precise. While few studies report a negative association between alcohol use and stroke [33,34], others show an increased risk of stroke with both acute [35] and chronic use of alcohol [36]. Our study decreased the odds of developing a stroke in respondents who use alcohol. Strong evidence shows a positive correlation between amphetamine use, smoking cigarettes, and stroke development [37-43]. Unexpectedly, our study showed a negative correlation between the use of amphetamines, alcohol, illegal injectable drugs, smoking cigarettes, and stroke development. The reason for this is unclear and could be related to our study's limitations, as mentioned below.

The temporal link between the stroke and the SUDs could be determined because both were examined simultaneously. Also, we could not distinguish primary from secondary or recurrent strokes; consequently, the incidence rates and population-attributable risk percentages refer to all strokes. The vast differences in results among various studies can be attributed to the misclassification of drug abuse history. Recall bias is another limitation of our study.

## Conclusions

Additional focused epidemiological studies and longitudinal studies are needed to assess the extent of the SUD's presence among the stroke population and distinguish primary from secondary or recurrent stroke among the SUD population. There needs to be more awareness about the rise of stroke in younger population groups and the modifiable risk factors, including the association between SUDs and stroke. Stroke victims could suffer from fatigue, anxiety, depression, and cognitive impairment affecting their day-to-day life.

Innovative and dedicated evidence-based public health procedures will allow practical steps to prevent and shorten the duration of illness in substance users and treat SUDs and their complications, such as stroke and infectious diseases. Techniques like providing information about substance use risks and strategies to quit or reduce use and use-related risk behaviors at schools, primary care, and mental health clinics should be prioritized. 
